# A Conserved Pre-Block Interaction Motif Regulates Potassium Channel Activation and N-Type Inactivation

**DOI:** 10.1371/journal.pone.0079891

**Published:** 2013-11-13

**Authors:** Paul J. Pfaffinger

**Affiliations:** Department of Neuroscience, Baylor College of Medicine, Houston, Texas, United States of America; Weizmann Institute of Science, Israel

## Abstract

N-type inactivation occurs when the N-terminus of a potassium channel binds into the open pore of the channel. This study examined the relationship between activation and steady state inactivation for mutations affecting the N-type inactivation properties of the *Aplysia* potassium channel AKv1 expressed in *Xenopus* oocytes. The results show that the traditional single-step model for N-type inactivation fails to properly account for the observed relationship between steady state channel activation and inactivation curves. We find that the midpoint of the steady state inactivation curve depends in part on a secondary interaction between the channel core and a region of the N-terminus just proximal to the pore blocking peptide that we call the Inactivation Proximal (IP) region. The IP interaction with the channel core produces a negative shift in the activation and inactivation curves, without blocking the pore. A tripeptide motif in the IP region was identified in a large number of different N-type inactivation domains most likely reflecting convergent evolution in addition to direct descent. Point mutating a conserved hydrophobic residue in this motif eliminates the gating voltage shift, accelerates recovery from inactivation and decreases the amount of pore block produced during inactivation. The IP interaction we have identified likely stabilizes the open state and positions the pore blocking region of the N-terminus at the internal opening to the transmembrane pore by forming a Pre-Block (P state) interaction with residues lining the side window vestibule of the channel.

## Introduction

Inactivation is an autoinhibitory process of ion channels that limits pore function in response to sustained depolarizations [1], [2], [3], [4]. N-type inactivation is one of the basic inactivation gating mechanisms of voltage-gated potassium channels [5]. In N-type inactivation, the cytoplasmic N-terminus of certain potassium channel pore forming or auxiliary subunits enters the pore of the open channel and blocks potassium ion conduction [6]. Under conditions where the channel is fully activated, the binding of the N-terminus into the pore is considered to be largely voltage-independent. The voltage dependence of the N-type inactivation gate comes from channel activation which determines the availability of the N-terminal binding site in the open channel [5].

**Figure 1:**
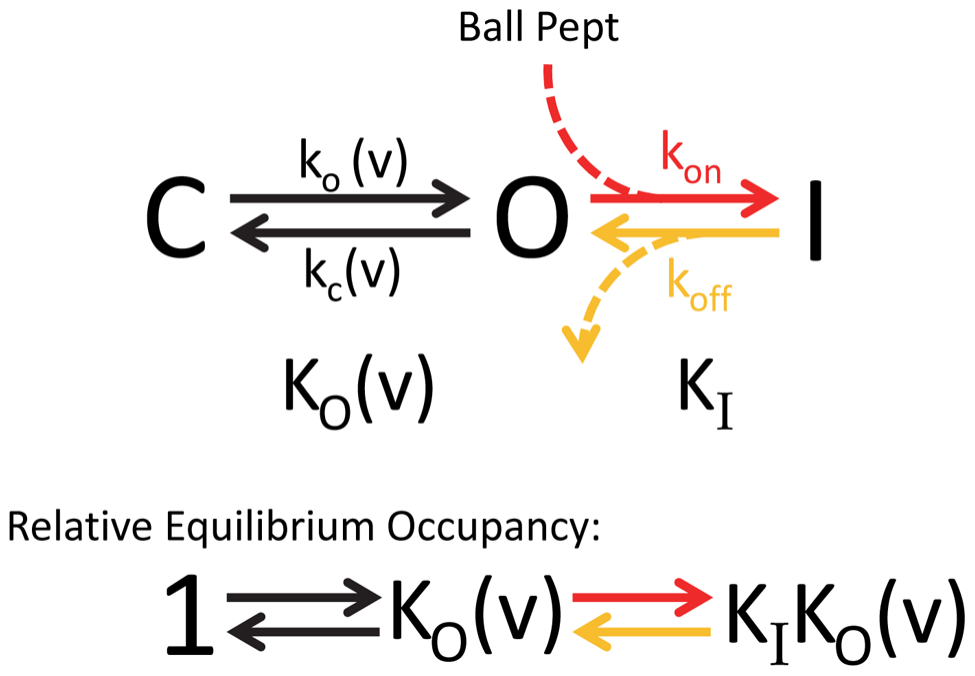
*Drosophila Shaker* based Single-Step Model for N-type Inactivation. Model has 3 basic states: Closed (C), Open (O) and Inactivated (I). Relative occupancy of these three states at equilibrium is given by the Equilibrium constants: Voltage-Dependent Activation- K_O_(v), and Inactivation- K_I_. The rate limiting step for inactivation at strong positive potentials is the binding of the N-terminus into the pore, the k_on_ rate, highlighted in red. The rate limiting step for recovery at strong negative potentials is the unbinding of the N-terminus from the pore block site, the k_off_ rate, highlighted in orange. These same rate limiting steps are proposed to control Ball Peptide inactivation (Dashed), or for that matter pore block by long chain quaternary ammonium derivatives like C9. Note that the rate limiting steps are not inherently voltage dependent; however, some voltage dependence to the recovery rate could be due to electric field effects mediated in the pore by K^+^ ions or charges on the ball peptide that accelerate unblocking, or re-blocking before closing that becomes less likely as k_c_(v) becomes faster.

Classically, N-type inactivation was modeled as a simple single-step reaction between the N-terminus and the open state of the channel [1], [5], [7], [8] (Fig. 1). In this “ball and chain” model, the tethered N-terminus diffuses freely below the pore until voltage-dependent gating opens the pore revealing the ball binding site. The pore blocking ball then binds to the block site at a rate limited by the time taken to diffuse from the swept volume into the pore. Recovery occurs as the unblocked channel rapidly closes at negative potentials following the slow unbind of the ball from its binding site. In the Classic Single-Step Model of N-type inactivation the affinity of the N-terminus for the pore binding site determines the fraction of current that is blocked during the inactivation reaction.

Perhaps the biggest conceptual problem with the Single-Step Inactivation model is to reconcile it with structural models of the channel [9], [10]. These structural models suggest a long and tortuous pathway before the ball peptide finally reaches its binding site just below the selectivity filter with multiple potential interaction sites present between the free and pore blocked states [11], [12]. Obviously, at some level the movement of the N-terminal peptide from a state of “free” diffusion in the cytoplasm in the closed state of the channel to pore blocking the open state must involve many different conformations that could be considered distinctive states. However, the need for a multi-step description of N-type inactivation requires first clearly showing that the N-type inactivation process cannot be explained by a single-step mechanism.

Some more recent studies have supported models for N-type inactivation involving the addition of one or more steps prior to the terminal pore-block reaction [11], [12], [13], [14], [15]. Zhou et al. (2001) proposed a two-step inactivation model for inactivation produced by a beta subunit N-terminus, where a region near the N-terminus first binds near the pore (Pre-Inactivation Step) before the N-terminal blocking peptide enters and blocks the pore (Inactivation Step) [13]. The Pre-Inactivation step was proposed as a way to explain why mutations at the N-terminus had large effects on recovery but not on the inactivation rate, whereas mutations further from the N-terminus affected both rates. In this model, the rate limiting steps for Inactivation and Recovery are the formation and loss of the Pre-Inactivated State not the terminal Inactivation step.

A problem with the two-step Inactivation model of Zhou et al. (2001) is that it suggested that terminal inactivation step involves rapid block and unblock of the pore, in contrast to the typically observed slow transitions, which appear to agree more closely with the Single-Step model [5], [16]. On the other hand, recent studies on BK channels have shown that rapid block-unblock does occur, at least with some N-type inactivation domains [15]. Finally, the significant voltage dependence to the inactivation Recovery kinetics is difficult to explain with either the single-step or two-step inactivation model as originally proposed. This discrepancy has led Camacho (2008) to propose a very clever tugging model where gating movements during channel closing tug the N-terminus from the pore [17].

To better understand the N-type inactivation process, we have been studying the *Aplysia* AKv1 potassium channel [18]. This channel has an N-type inactivation domain that is well conserved in Kv1 channels over a wide range of species [11]. The AKv1 N-type inactivation reaction appears to involve a series of distinct steps. In this channel, the rate limiting transition for N-type inactivation at strong depolarizations appears to involve a series of conformational changes where the electrostatic attraction of a highly charged Polar region of the N-terminus to the channel core facilitates the transfer of the N-terminus into the side window openings [11], [12]. Analysis of tail currents suggested that significant conductive and non-conductive states also exist after the rate limiting transition step is passed [11], [12]. Because the Pre-Inactivated state is closely associated with the rate limiting step of the specific two-step inactivation model of Zhou et al. (2001) [13], and appears more synonymous to the initial electrostatic attraction of the Polar region, the two classes of states that exist after the rate limiting step were named Pre-Block and Pore-Block states [12]. Finally, these studies suggested that channel closing might be the rate limiting step for recovery, rather than disruption of a “Pre-Inactivated” state, with only the Pore-Blocked state delaying recovery [11], [12]. Importantly, if Pore block/unblock is fast, this model can explain the similar voltage dependence between recovery and normal channel closing without requiring the spring based mechanism proposed by Camacho.

For mutants of AKv1 that increase the hydrophobicity of the N-terminus, it seems clear that at least some of the Pre-block states involve interactions of the N-terminus with other parts of the channel along the inactivation pathway but outside the pore [11]. However, the extent to which such interactions normally occur during the inactivation of the wild type channel is unclear. In this study we sought to determine if specific Pre-Block binding interactions mediated by the N-terminus are occurring after the initial electrostatic attraction between the N-terminus and channel occurs and whether these interactions enhance or deter N-type inactivation. Our results here show that AKv1 channels form a clearly delineated Pre-Block state (P State) separate from the Pore-Blocked state. The P state is formed by a region of the N-terminus proximal to the pore blocking Inactivation Ball. The formation of the P state produces a channel with shifted activation properties and enhanced N-type inactivation. Disruption of the P state interactions by mutagenesis greatly reduces the ability of the N-terminus to inactivate the channel. Unlike the classic Pre-Inactivation Model, we find that disruption of the P state has little effect on the time course of inactivation at strong depolarizations.

## Results

### *Drosophila* Single-step N-type Inactivation Model poorly predicts steady state Inactivation for AKv1

To better understand the importance of a multiple-state model for describing the N-type inactivation properties of the AKv1 channel we first sought to determine how the equilibrium inactivated state of the channel differs from the predictions of a single-step inactivation process. To address this question, we first need to consider the predictions of the classic single-step N-type inactivation model (Fig. 1) with voltage-dependence due to channel activation [5], [16], [19]. Although channel activation is a complex multi-step process, we can approximate the voltage-dependence for channel activation using an apparent Activation equilibrium constant based on the single Boltzmann activation curve fit parameters as:



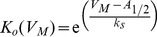
(1.1)


where A_1/2_ is the half activation potential and k_s_ is the slope factor. For the *Drosophila* N-type Inactivation Model shown in Fig. 1, inactivation is voltage dependent due to activation voltage-dependence and will follow a Boltzmann curve, which we can plot normalized by the maximum possible inactivation as (see Methods):
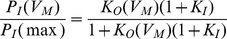



At the half inactivation midpoint potential, I_1/2_, the term on the right is equal to ½, so obviously: 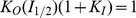



This equation can be linearized by substituting in Equation (1.1) and taking the natural log:



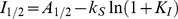
(1.2)


So a key prediction of the classic single-step N-type Inactivation Model is that if we plot I_1/2_ as a function of ln(1+K_I_) for a number of inactivation ball mutants with different inactivation equilibrium constants, the data should fall on a straight line with slope –*k_S_* and y-intercept *A_1/2_*. We have previously generated a number of variants of the AKv1 channel by mutating residue 2, creating the AKv1(E2x) series (x =  E, D, T, A, Q, N, K). These mutants display a range of inactivation properties, including dramatically different levels of fractional block during N-type inactivation [11]. We used these mutants to determine how well a single-step model predicts the steady state inactivation properties of the channel. In order to reduce complications due to C-type inactivation, recordings were performed in elevated external potassium which reduces C-type inactivation to less than 5% over a 1 sec depolarization. Finally, we will use steady state voltage-dependent gating properties as a reasonable estimate for the equilibrium inactivated state of the channel.

**Figure 2:**
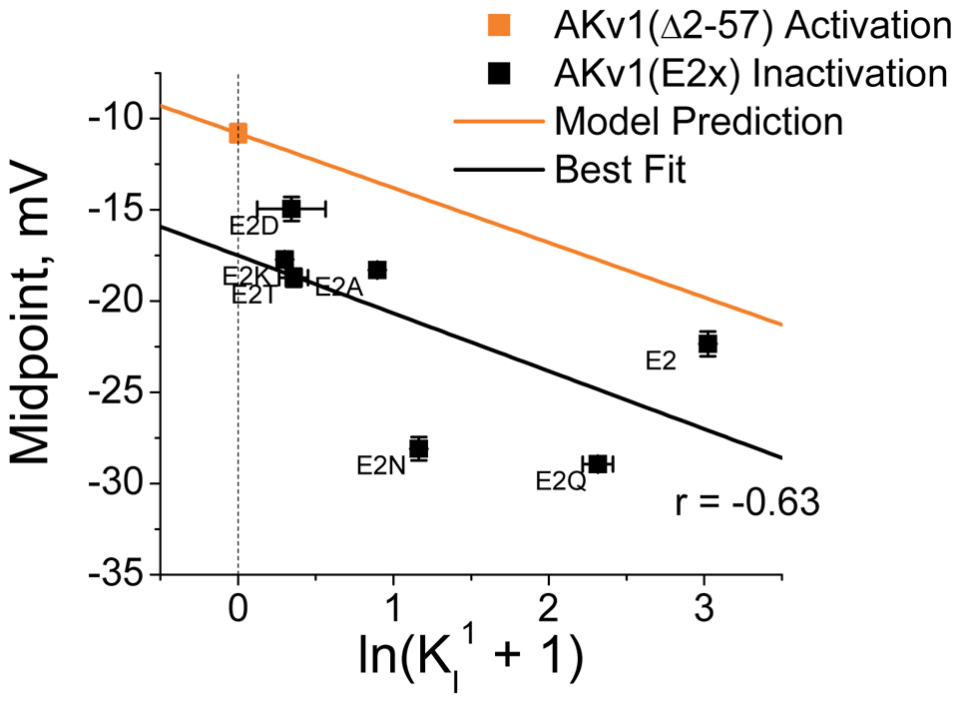
*Drosophila Shaker* based Model Poorly Predicts AKv1 Steady State Inactivation. Best fit to AKv1(E2x) channel data is shown in black. While there is a trend in the data matching the prediction of the model, error bars for most data points do not contact the best fit line. The y-intercept of the best fit is significantly different from the activation midpoint for AKv1(Δ2-57).

The baseline activation properties of the AKv1 channel were measured using an N-terminal deletion mutant where the entire “ball and chain” region before the N-terminus has been deleted, AKv1(Δ2-57) (A_1/2_ = −10.8 ± 0.5 mV (10); k_s_ = 2.9 ± 0.1 mV(10)). Fractional block was measured at strong positive potentials for the AKv1(Ex2) mutants to estimate K_I_ for the single-step inactivation reaction shown in Fig. 1 (K_I_^1^ measure). Next, steady state inactivation curves were measured for the different AKv1(E2x) channels and the midpoint plotted as a function of ln(1+ K_I_^1^) according to Equation (1.2) (Fig. 2). While we see the expected trend in the data (r = −0.63), the best fit line shows that the data error bars generally do not cross the trend line, meaning this data set does not align in a linear manner as expected for this model. Compared to the prediction from a model based on the activation properties of AKv1(Δ2-57), all the data points plot to the left of the expected curve as if K_I_^1^ is underestimating the real K_I_ value.

### Testing Framework with Computational Models

**Figure 3:**
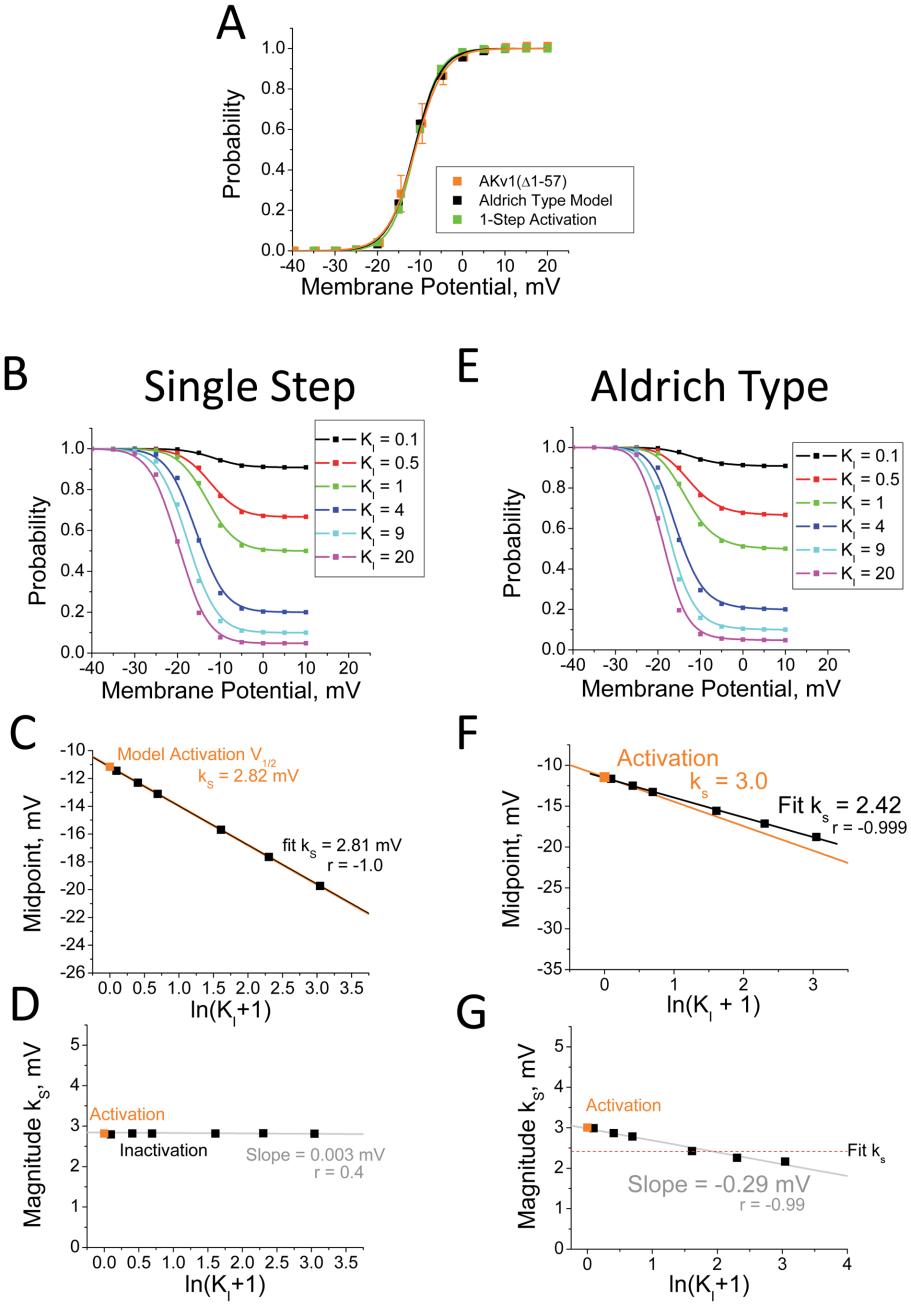
Comparing Effects of more Complex Activation Models on Steady State Inactivation Properties. Single-Step Activation Model for AKv1 was compared to channel activation model based on Zagotta et al.(1994), slightly modified to match AKv1 gating (see Methods).[20] A) Model Parameters were adjusted to optimally match steady state activation curve for AKv1(Δ2-57). B) Single-Step Voltage-dependent activation model was combined with a single-step Inactivation Model with varying K_I_. C) Steady State Inactivation midpoint matches prediction from the activation properties for the model. D) Voltage dependence for steady state inactivation matches activation k_s_, and is not affected by varying K_I_. E) Zagotta et al. (1994) based activation model [20] adjusted for AKv1, was combined with a single-step Inactivation Model with varying K_I_. F) Data fit well to a linear model with slope slightly less than predicted by channel activation (orange). G) Flatter slope for fit k_s_ is caused by greater voltage dependence as inactivation is shifted to more negative potentials, as expected due to reduced occupancy of intermediate closed states at more negative potentials.

The deviations of the data in Fig. 2 from the predicted curve might indicate a problem caused by a simplified single-step activation model, rather than a problem in modeling inactivation as a single-step process. We therefore used computational modeling to compare the effects of switching from a single-step activation model to a more accurate activation model for the AKv1 channel based on the Aldrich group’s studies on *Drosophila* Shaker channel activation [20]. Using QuB, single-step and Aldrich style activation models of AKv1(▵2-57) were made that approximate the kinetics and voltage-dependent activation properties of the AKv1 channels (see Methods) (Fig. 3A). We then determined the effects of adding a single-step N-type inactivation step to both models with K_I_ varying from 0.1 to 20 (Fig. 3B, E). Data values were simulated using QuB and inactivation curves were generated by fitting with Boltzmann functions to determine slope factor and half inactivation values.

Using the single-step activation model, plotting the half inactivation values as a function of ln(1+K_I_) produces a linear relationship (r = −1.0) where the measured slope is very close to the negative of the activation slope factor k_s_ and the y-intercept passes exactly through the measured half activation value (Fig. 3C). For the measured inactivation curves, the slope factor values are essentially identical to the activation slope factor regardless of the K_I_ value (Fig. 3D). Interestingly, the inactivation midpoint data for the Aldrich type activation model also fit well to a linear regression line (r = −0.999) and the y-intercept intersects the midpoint for the activation curve as predicted (Fig. 3F). However, two significant differences were seen from the single-step activation model: 1) The slope of the fit line predicts a value for k_s_ that is quite a bit smaller than the measured activation curve slope factor (Fit k_s_ = 2.42 mV; Activation k_s_ = 3.0 mV), and 2) The slope factor determined from the inactivation curve fits got smaller as the K_I_ value increased (Fig. 3G). Both of these changes make sense when considered in the context of the model. The activation curve for the Aldrich model is less steep than the overall voltage dependence between fully closed and fully opened due to the voltage-dependent occupancy of intermediate closed states. As the inactivation curve shifts to more negative potentials, intermediate states become less occupied and more of the overall voltage dependence is determining the open probability, hence the slope factor gets smaller. The key result from this modeling is that the inactivation midpoint shifts in a predictable manner when K_I_ is changed regardless of the complexity of the activation model used. We conclude, therefore, that the wide scattering of the data in Fig. 2 from the prediction line is due to a failure in the single-step inactivation model not in a failure to adequately model channel activation.

### Relationship between Recovery from Inactivation and Channel Closing

It seems logical that the binding affinity of the N-terminus for the pore should somehow be related to the equilibrium fraction of channels in the inactivated state. The single-step *Drosophila* model assumes that the channel is blocked whenever the N-terminus is beyond the rate limiting step. Therefore, the most logical conclusion from the results thus far is that this assumption is wrong. Instead it seems most likely that the K_I_^1^ measure is a lower estimate for the pore binding affinity of the N-terminus due to the presence of conductive states where the N-terminus is beyond the rate limiting step, but not blocking the pore, as previously hypothesized [11], [12].

Previous studies have suggested that potassium channels cannot close if the N-terminus is within the pore [1]. This effect produces extended tail currents during recovery as the channel passes back through the open state before closing and thus excluding the N-terminus from the pore. For a channel with N-type inactivation, Recovery from inactivation at large negative potentials can be simplified to the following reaction scheme:



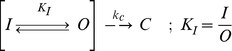
(Recovery Gating Scheme)


where *k_c_* is rate for the essentially irreversible (at strongly negative potentials) closing reaction for a channel without N-type inactivation. In this model, the kinetics for Recovery are determined by the rate limiting step which could be either the rate to unblock (*I* →*O*) or to close (*O*→ *C*). Recovery should have no specific relationship to closing kinetics if the pore block-unblock is rate limiting, a variable relationship to closing if the block-unblock rates are similar to closing, but if pore block and unblock are rapid compared to closing then there should be a fixed relationship between the time constants to close and recover given by (see Methods):



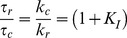
(1.3)


**Figure 4:**
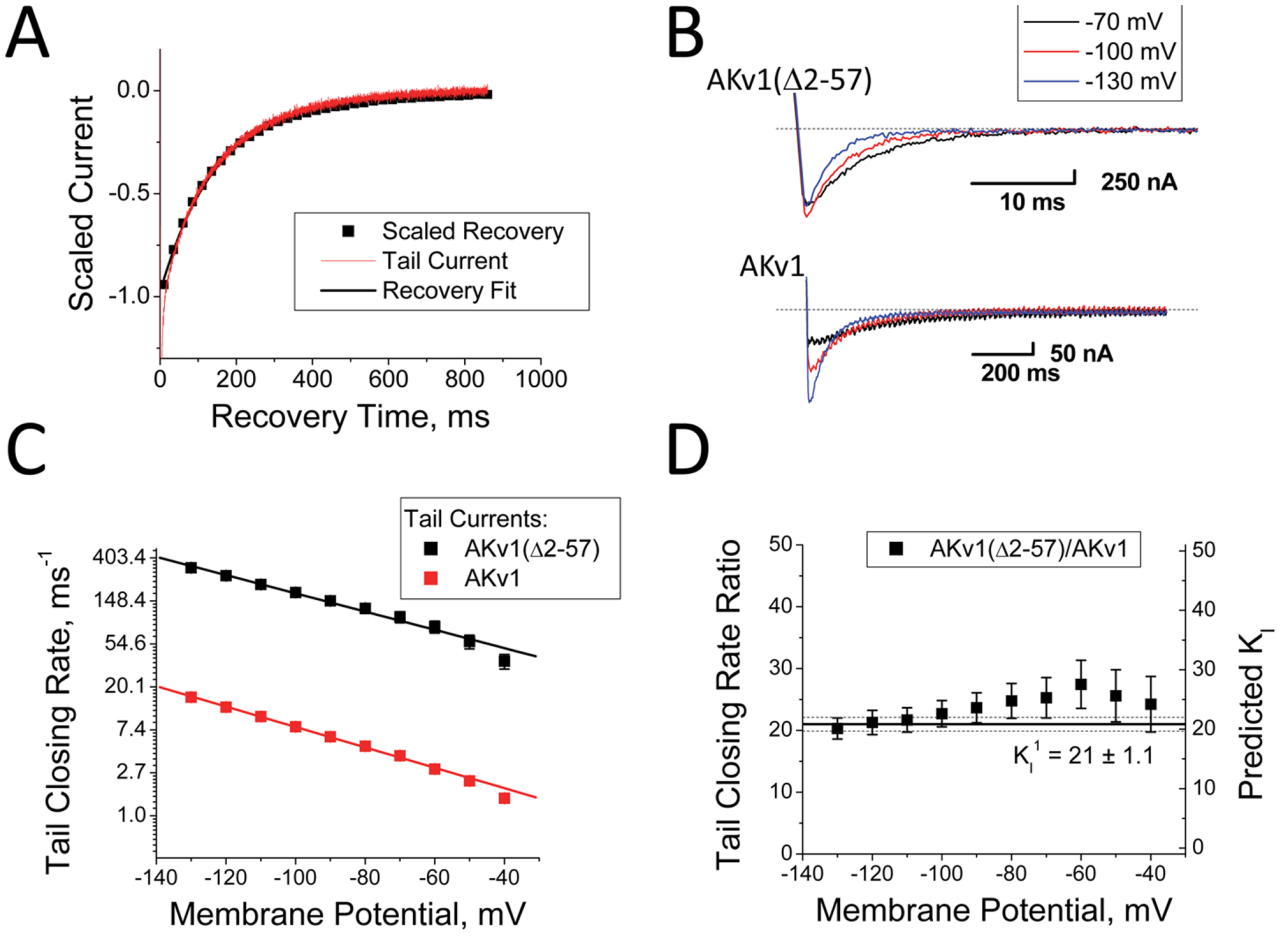
Slowed Closing produced by N-type Inactivation matches the voltage-dependence for normal channel closing. A) Time course for AKv1 tail currents matches the Inactivation recovery time course. B) Kinetics for normal closing measured in AKv1(Δ2-57) and recovery tails measured in AKv1 are voltage dependent. C) Comparing tail current closing rates from single exponential fits. Voltage-dependence for AKv1(Δ2-57) closing matches the voltage-dependence for AKv1 tail currents despite the dramatically different rates. D) Ratio of tail closing rates predicts a consistent value for K_I_. For wild type AKv1 this value is similar to K_I_^1^ measured from the fraction of current that is not inactivated at the end of a pulse.

To determine the relationship between recovery kinetics and channel closing kinetics at different potentials we compared closing kinetics for AKv1(Δ2-57), which lacks N-type inactivation, to the tail currents for AKv1, which match recovery kinetics (Fig. 4A). Although the tail currents for AKv1 are much slower than normal channel closing (Fig. 4B), the voltage-dependence of the closing and recovery time constants given by the slopes on a semi-log plot are very similar (Fig. 4C), and the ratio provides a consistent estimate for K_I_ (Fig. 4D). For AKv1, this estimate for K_I_ based on the rate of slowing of channel closing is similar the K_I_^1^ estimate based on fraction block at strong depolarizations (+50 mV: 0.955 ± 0.002 (22)) gives a K_I_^1^ of 21 ± 1.1.

It is important to note that this analysis does not prove that the K_I_ reaction kinetics are fast, since other models using different mechanisms to couple recovery and closing, such as the Camacho model, can produce a similar result [17]. Rather, the importance of this result is that this approach provides an estimate for K_I_ that does not depend on the number of steps leading up to the inactivated state, only on how the affinity of the terminal inactivation reaction affects channel closing. Equation (1.3) can be used to replace the term in the logarithm in Equation (1.2) to formulate a more general model to explain the midpoint of the steady state inactivation curve based on the slowing of channel closing during recovery:



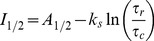
(1.4)


### Steady State Inactivation Midpoint is a Function of Slowing of Closing during Recovery

**Figure 5:**
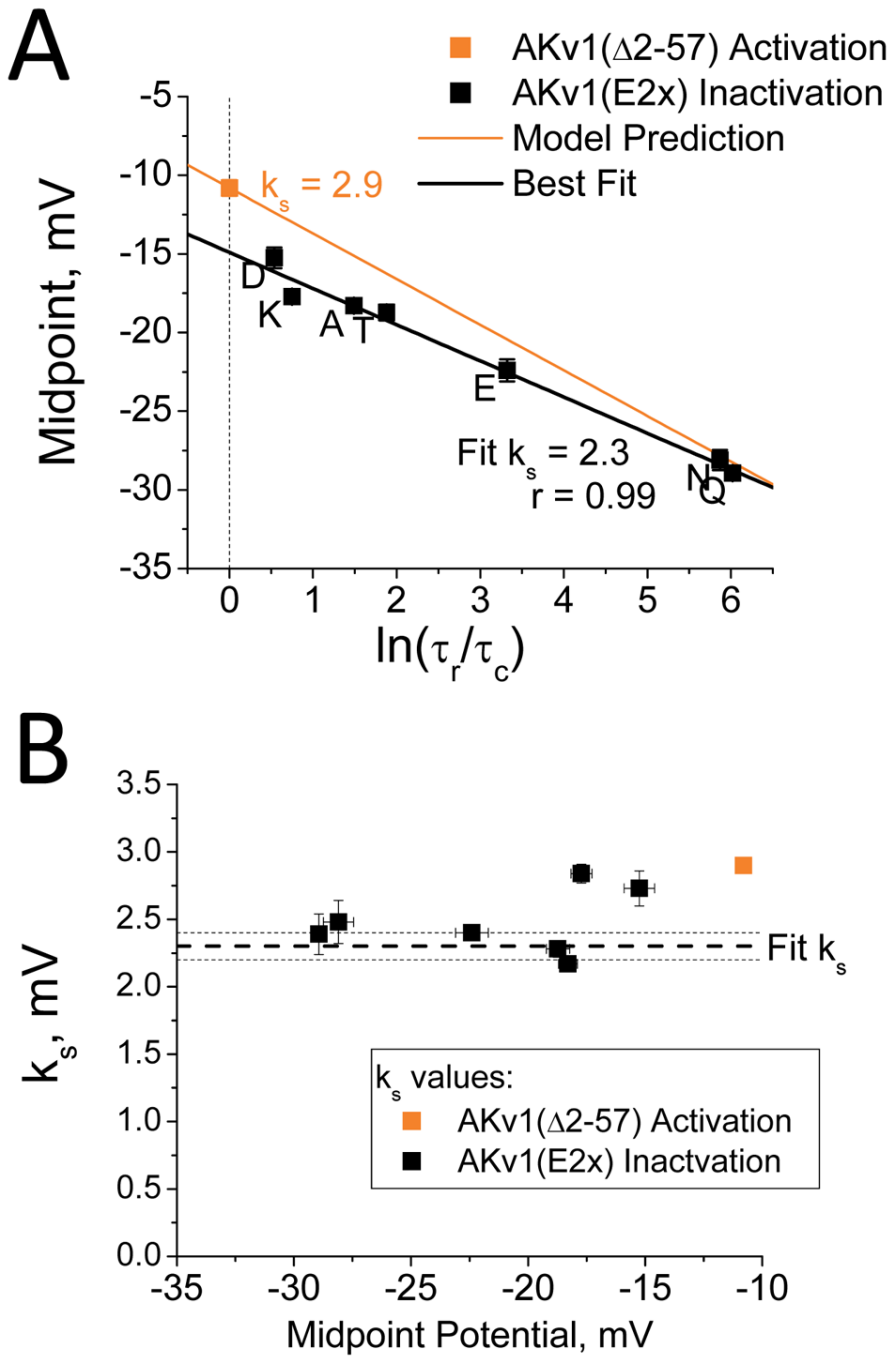
Slowed Closing predicts steady state inactivation midpoints for AKv1(E2x) series mutants. A) Best fit to AKv1(E2x) channel data is shown in black. Prediction for the steady state inactivation midpoints based on AKv1(Δ2-57) activation gating shown in orange. AKv1(E2x) data are well fit with a linear model (r = −0.99); however, the y-intercept is more negative than expected and the slope is flatter than predicted from AKv1(Δ2-57) activation. B) Predicted value for k_s_ from the fit is smaller than the activation curve k_s_, and similar to k_s_ values measured from the inactivation curves, as expected from the more complex multi-step activation of the real channel.

Based on Equation (1.4), we plotted steady state inactivation curve midpoints for the AKv1(E2x) mutants as a function of ln(τ_r_/τ_c_) (Fig. 5A). Unlike the situation found in Fig. 2 when using the K_I_^1^ estimate, the data for the AKv1(E2x) mutant series now plot as a very linear relationship as predicted (r = −0.99). However, the measured curve displays a significant negative shift in the y-intercept (−14.8 mV, P<0.0001) from the expected value equal for *A_1/2_*, based on the half activation value for AKv1(▵2-57). In addition, the slope of the measured curve is flatter than predicted, (k_s_ = 2.3 mV, P = 0.001), and as Fig. 5B shows, the *k_s_* values from Boltzmann fits to the steady state inactivation curves for most mutants are significantly smaller than found for steady state activation, and much closer to the slope from the fit to the data in Fig. 5A. It seems likely that the flatter slope measured in Fig. 5A and lower k_s_ values we measure for AKv1(E2x) mutant steady state inactivation curves reflect complexities of the activation process as found in the Aldrich Activation model (Fig. 3F,G). However, the offset of the y-intercept from *A_1/2_* in Fig. 5A is not explained, since neither Activation model had any effect on the y-intercept.

### N-terminal Domain Effects on Channel Activation

**Figure 6:**
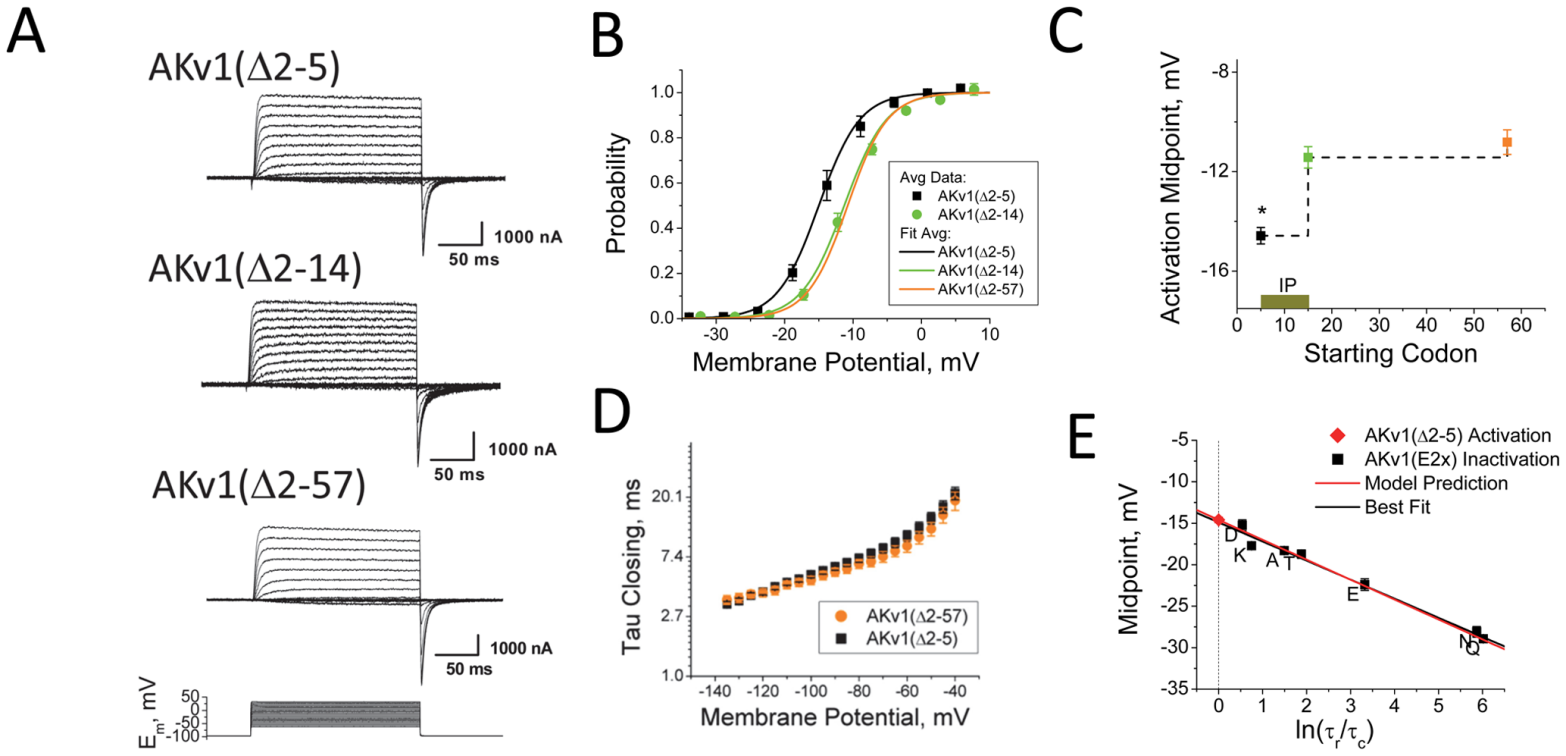
Identification the Inactivation Proximal (IP) Domain. A) Representative currents for N-terminal deletions. Removal of the initial 5 residues eliminates N-type inactivation. B) Activation midpoint for AKv1(Δ2-5) is shifted to a more negative midpoint compared to larger N-terminal Deletions AKv1(Δ2-14) and AKv1(Δ2-57). C) Deletion effects on activation midpoint identify the IP region between residues 5-14. D) Despite the shift in activation midpoint, AKv1(Δ2-5) closing kinetics and voltage dependence are similar to AKv1(Δ2-57). D) Using AKv1(Δ2-5) activation midpoint and slope from AKv1 inactivation accurately predicts inactivation midpoints for AKv1(E2x) mutant series (Model Prediction line).

The fact that the y-intercept in Fig 5A converges to a more negative value than the activation midpoint for AKv1(Δ2-57) strongly suggests that the underlying activation midpoint for a channel with an intact N-terminus is about 4 mV more negative AKv1(Δ2-57). To better understand how the N-terminus might affect channel activation gating, we constructed smaller N-terminal truncation mutations and examined their impact on activation and inactivation gating. Figure 6A compares the gating behavior the AKv1 channel as progressively larger segments of the N-terminus are removed. Whereas wild type AKv1 shows robust and rapid N-type inactivation, deletion of even a small region of the N-terminus, AKv1(Δ2-5) is sufficient to completely eliminate N-type inactivation (Fig. 6A). Progressively larger deletions, AKv1(▵2-14) and AKv1(Δ2-57), had no additional effects on inactivation gating, identifying the initial N-terminus as the critical motif for pore block (Fig. 6A). We define the minimal region required for pore block that is disrupted in the AKv1(Δ2-5) truncation the **I**nactivation **B**lock (**IB**) region.

Despite the similar elimination of N-type inactivation gating for all three truncations, we identified an interesting activation difference between AKv1(Δ2-5) and the larger two deletions. Steady state activation curves show that activation for AKv1(Δ2-5) (−14.6 ± 0.3 mV (8), P<0.0001) is significantly shifted towards more negative potentials compared to AKv1(▵2-14) (−11.3 ± 0.4 mV (6)) and AKv1(Δ2-57), which are not significantly different from each other (P = 0.5) (Fig. 6B). Neither deletion produces a significant change in activation voltage-dependence compared to AKv1(Δ2-57): (AKv1(Δ2-5): *k_s_* = 3.1 ± 0.1 mV (8), P = 0.18, NSD. AKv1(▵2-14): *k_s_* = 3.2 ± 0.1 mV (5), P = 0.08, NSD). These results suggest that an activation modulating region exists on the N-terminus that is functional in AKv1(Δ2-5) but disrupted in AKv1(▵2-14) (Fig. 6C). We have named the (5-14) region that is required for this shift in activation the **I**nactivation **P**roximal (**IP**) region due to its position just following the IB region. Despite the shift in activation, closing kinetics for AKv1(Δ2-5) are very similar to other non-inactivating AKv1 channels (Fig. 6D), likely because the IP region does not bind in the pore, as opposed to the IB region, which does slow channel closing.

We next compared the activation midpoint for AKv1(Δ2-5) to the y-intercept for the best fit to the AKv1(E2x) data from Fig. 5A, and find that the two are indistinguishable (P =  0.56, NSD) (Fig. 6E). In addition, as suggested by the Aldrich type model, if we use the value for *k_s_* obtained from AKv1 channel steady state inactivation then we closely predict the midpoints for steady state inactivation for the entire AKv1(E2x) mutational series. We conclude, therefore, that the identified IP region is fully responsible for the anomalous shift seen in these measurements.

### A Conserved Motif Required for the IP region’s effects on Activation

**Figure 7:**
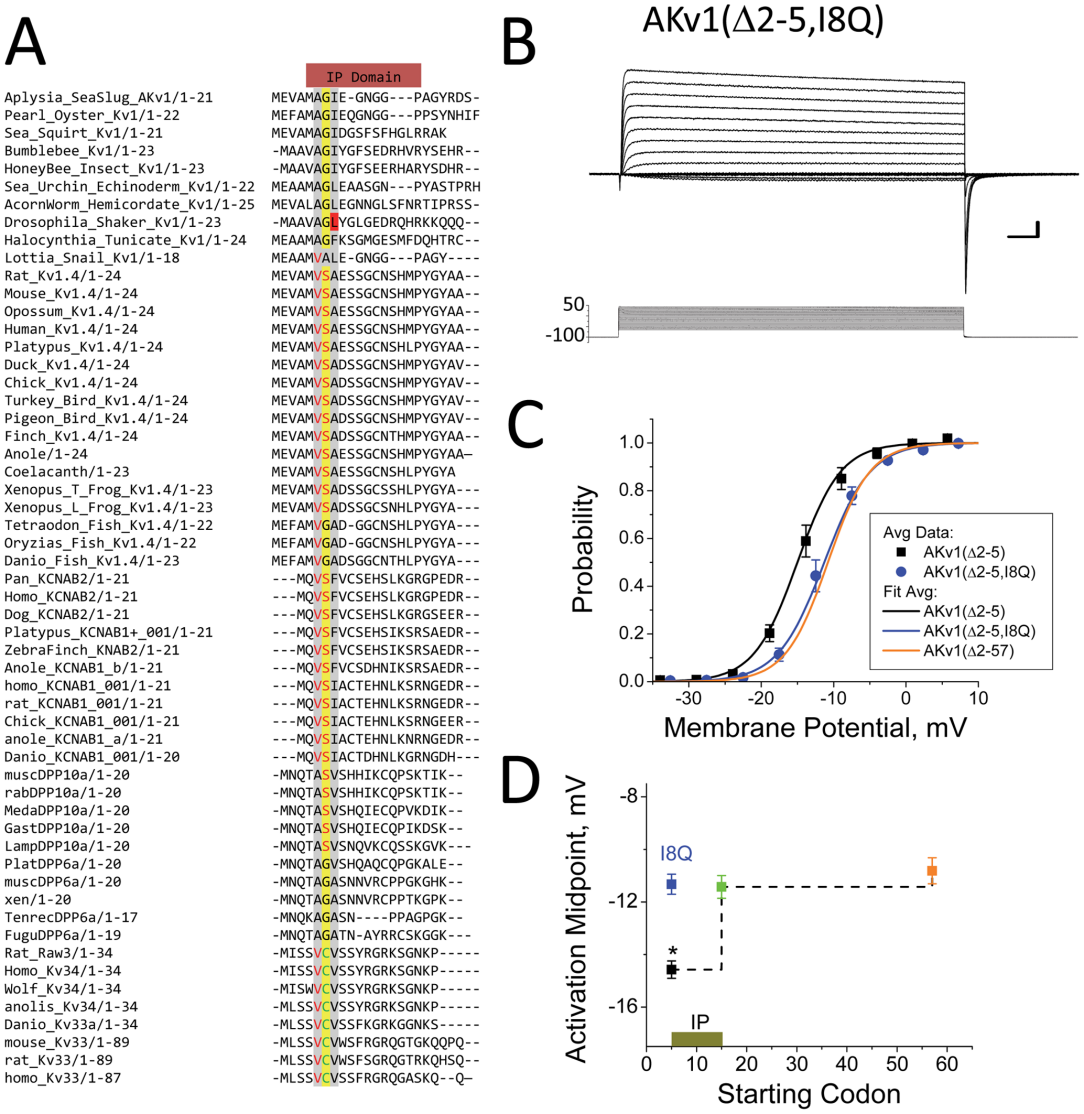
Identification of Conserved Motif in IP Domain. A) IP Domain retained in AKv1(Δ2-5) and deleted in AKv1(Δ2-14) contains a highly conserved [(A/V)-(G/S/C)-(H_5_)] Motif. Mutations to residue Leu7 from *Drosophila* ShB channel, highlighted in red, that make this residue more polar disrupt ShB N-type inactivation. B) AKv1(Δ2-5, I8Q) mutant shows expected non-inactivating currents. C) Activation Curve for AKv1(Δ2-5, I8Q) is shifted back to more positive potentials and matches AKv1(Δ2-57). D) Summary data showing deletions and mutations identifying the IP Domain and the disruption of IP Domain effects on activation by the I8Q mutation.

To identify key IP region residues we aligned N-type inactivation domains from a number of different potassium channels and potassium channel auxiliary subunit proteins (Fig.7A). We identified a tripeptide motif present in the IP region (IP motif) that is surprisingly evident in a large number of N-type inactivation domains [(A/V)-(G/S/C)-(H_5_)], where H_5_ is one of 5 different hydrophobic amino acid (I, L, F, A, V). This sequence similarity probably also reflects convergent evolution since the N-type inactivation domains from different gene families likely evolved separately. Interestingly, previous studies on *Drosophila* Shaker channel inactivation showed that mutations to Leu7, the H_5_ residue in this N-terminus (Fig. 7A, red highlight) strongly disrupt N-type inactivation when changed to a polar or charged residue [5], [8].

Of the 9 residues contained in the IP region, 5 are structural (4-Gly, 1-Pro) and therefore poor initial targets for mutagenesis, leaving 4 residues available to potentially target by mutagenesis: Ala6, Ile8, Glu9 and Asn11. Previous mutagenesis studies on Glu9 showed that interactions of the N-terminus with the channel, most likely within the side window vestibule, increase as this residue is made more hydrophobic [11]. The only strongly hydrophobic residue in the IP region is Ile8 (Estimated side chain burial kcal/mol: Ile 2.7, Ala 1.0, Glu 0.5, Asn -0.1) [21]. Given this observation, along with the fact that Ile8 is within the IP motif, and the obvious effects of mutagenesis of the homologous *Drosophila* residue Leu7, we decided to mutate Ile8 to test if this residue plays an important role in IP region function. To simplify our analysis we excluded charge changing mutations since separate electrostatic interactions between the N-terminus and the side window openings to the pore have a big effect in setting the inactivation time course [11], [12]. Asparagine and glutamine are the most polar uncharged amino acids, and glutamine is closer in size to isoleucine, so the mutation I8Q was introduced into the AKv1(Δ2-5) channel to make AKv1(Δ2-5, I8Q). Voltage-clamp analysis shows that the AKv1(Δ2-5, I8Q) channel has no fast inactivating component (Fig. 7B), as expected, but the activation midpoint (-11.3 ± 0.4 mV (5), P<0.0001) is shifted to more positive potentials compared to AKv1(Δ2-5) and not significantly different from AKv1(Δ2-57) (P = 0.58, NSD) (Fig. 7C). Again, there was no significant difference in voltage dependence produced by the I8Q mutation (*k_s_* = 3.2 ± 0.1 mV (5), P = 0.52, NSD). We conclude that the I8Q mutation completely disrupts the ability of the IP region to affect the channel activation midpoint (Fig. 7D).

### Role of IP region in N-type Inactivation

**Figure 8:**
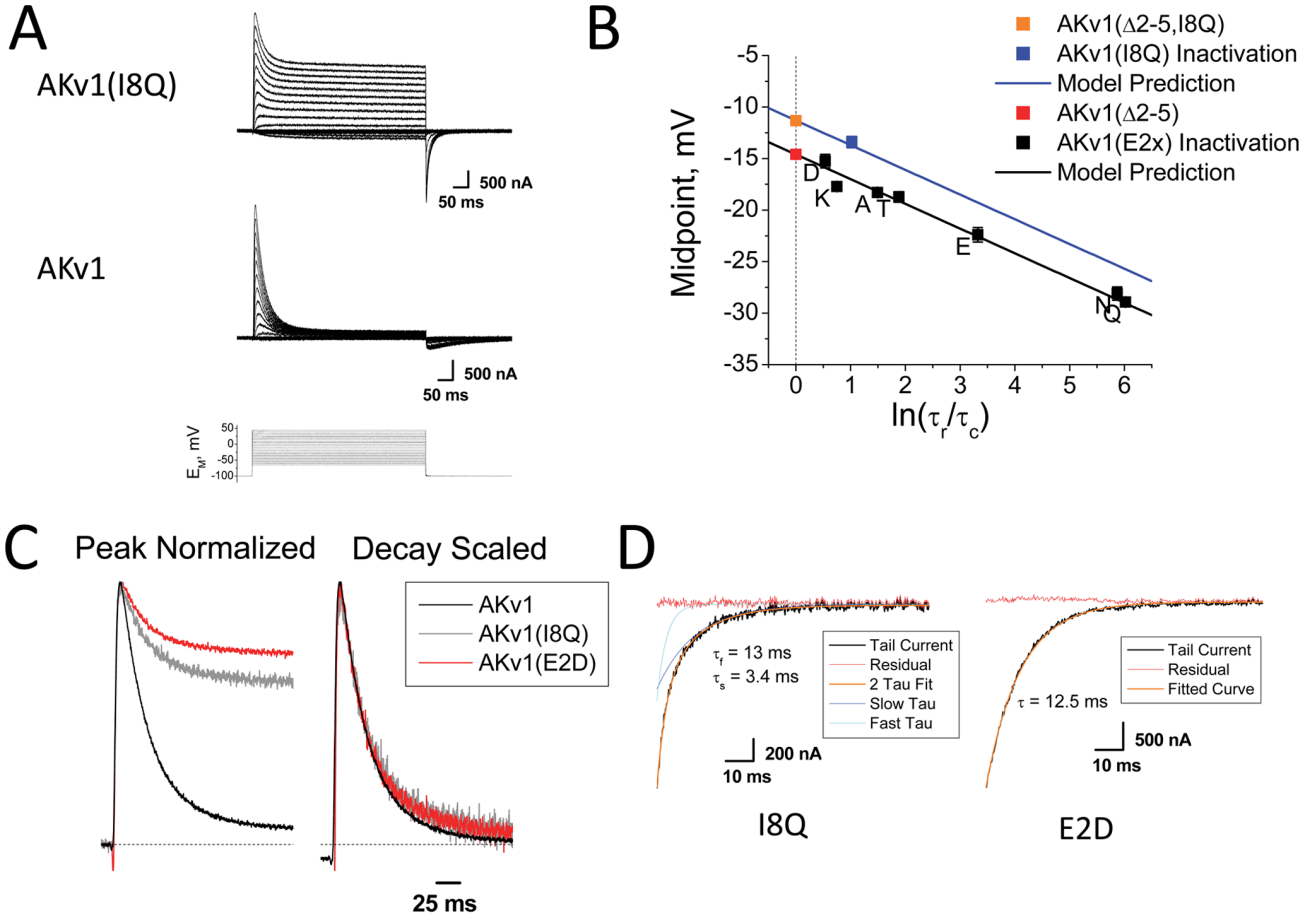
I8Q mutation has distinct effects on AKv1 Inactivation. A) Inactivation of AKv1(I8Q) is much less complete that AKv1, similar to the IB region mutant AKv1(E2D). B) Inactivation midpoint for AKv1(I8Q) is predicted by the activation midpoint for AKv1(Δ2-5, I8Q) further showing that the regulation of activation by the IP region is retained during N-type inactivation of AKv1(E2x) channels. C) Despite less efficient inactivation block, the kinetics for inactivation of AKv1(I8Q) and AKv1(E2D) are not significantly different from AKv1. D) Despite similar inactivation levels and inactivation and recovery time courses, tail currents for AKv1(I8Q) show 2-exponential decay whereas AKv1(E2D) tail currents are single exponential.

To better understand the role of the IP region in N-type inactivation, we inserted the I8Q mutation into the wild type AKv1 channel and examined its effects on N-type inactivation. Current traces show that the IQ8 mutation dramatically decreases the fraction block at strong depolarizations from 0.955 ± 0.002 (22) to 0.48 ± 0.02 (6), P<0.0001 (Fig. 8A) and accelerates the recovery from inactivation (Recovery −100 mV: 15.3 ± 0.6 ms (6), P<0.0001). The half inactivation voltage for AKv1(I8Q) also shows a strong positive shift compared to wild type (−13.4 ± 0.6 mV (6), P<0.0001) with no significant different in voltage-dependence (*k_s_* = 2.6 ± 0.14 mV (6), P = 0.78, NSD). To determine if these values plot with the AKv1(E2x) mutant series, we replotted this data as in Fig. 5A and compared the results for the expectation if the shift in activation produced by the IP region is eliminated (Fig. 8B). It is clear from this plot that the steady state inactivation for AKv1(I8Q) is shifted to positive potentials compared to the AKv1(E2x) curve, and the midpoint value is non-significantly different from the predicted value based on a channel lacking the IP region activation shift.

Despite the dramatic decrease in fractional pore block, the I8Q mutation had little impact on the inactivation time course. Wild type AKv1 inactivation kinetics at strong depolarizations (+50 mV: τ_I_ = 30.3 ± 1.2 ms (29)) are not significantly different from AKv1(I8Q), (+50 mV: τ_I_ = 26.5 ± 1.3 ms (6), P = 0.17, NSD) (Fig. 8C). This observation agrees with earlier studies on AKv1 that showed that the time course for inactivation is primarily determined by charge-charge interactions with residues near the side window openings to the channel, none of which are affected in the I8Q mutation [11], [12].

The inactivation and recovery kinetics of AKv1(I8Q) are very similar to the weakly inactivating IB region mutant AKv1(E2D) (+50 mV: *f*_I_ = 0.49 ± 0.5(6), τ_I_ = 25.6 ± 1.4 ms (6); −100 mV: τ_r_ = 11.6 ± 0.8 ms (4)) (Fig 8C). Despite these similarities, tail currents for these two mutants are strikingly different (Fig. 8D). Even though a majority of the current is not inactivated, AKv1(E2D) shows a single exponential tail current whose kinetics are similar to the inactivation recovery time course (−100 mV: τ = 11.6 ± 0.8 ms (4)). In contrast, the AKv1(I8Q) tail currents are clearly double exponential with a fast component that is slightly faster than the closing kinetics for non-inactivated channels (−100 mV: τ_f_ = 3.0 ± 0.7 ms (5), P = 0.03) and a slower component similar to the inactivation recovery time course (−100 mV: τ_s_ = 16.3 ± 1.5 ms (5), P = 0.54, NSD).

## Discussion

Our studies here have identified the IP region as a specific region of the N-terminus that shapes the inactivation process, but is not directly involved in pore block. It is clear that the IP region is capable of binding to a specific site in the channel in the absence of N-type inactivation because this binding is associated with a clear shift in the activation curve midpoint. Circumstantial evidence strongly suggests that the IP region forms an identical binding interaction during N-type inactivation since the steady state inactivation curve midpoint shows clear evidence of the same shift. Within the IP region we have identified a specific motif that is highly conserved across most N-type inactivation domains, even those that are likely produced by convergent evolution. By mutagenesis of the H_5_ residue of the IP motif to a more polar amino acid, we can effectively negate the IP region’s effects on channel activation and severely disrupt N-type inactivation. The energetics of the I8Q mutation strongly suggests that the H_5_ side chain is likely fully buried in a hydrophobic pocket in the channel. Hydrophobic burial of the Isoleucine side chain releases around 2.7 Kcal/mol of energy [21]. From the channel’s voltage dependence, the observed activation shift of 3−4 mV produced by Ile8 binding would require 0.7−1.0 Kcal/mol energy input. For inactivation, the observed change in fractional block produced by Ile8 binding is from 0.48 to 0.955 requiring an energy input of around 1.8 Kcal/mol, accounting for all the remaining binding energy produced by complete hydrophobic burial of the Ile8 side chain.

Although the IP motif is clearly associated with the IP region’s binding to the channel, it is not clear that this binding is the only factor that explains its conservation. Based on the likely location of the IP binding site just outside the pore inner vestibule in the side window vestibule, it is likely that the N-terminus must be sufficiently flexible in this region to allow a near right angle turn from the side window vestibule into the pore. Indeed, our original studies on AKv1 identified a glycine rich region contained with the IP region that was called the Flex region [12]. Regardless of whatever additional roles the IP motif plays, the ability to bind and form a Pre-Block interaction with the channel core has important consequences for channel activation and inactivation gating.

### How the IP-region regulates N-type Inactivation

**Figure 9:**
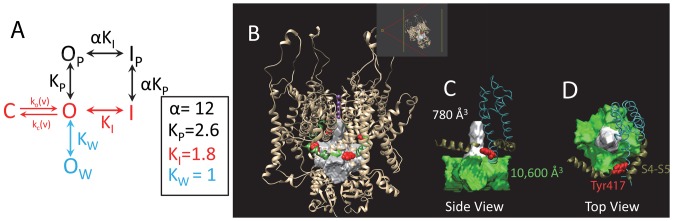
Modeling the Pre-Block Interaction. A) Minimal AKv1 N-type Inactivation gating scheme. AKv1 gating model is only slightly more complicated than the Single Step *Drosophila* Model (red states) because in addition to the **C**, **O** and **I** labels to indicate the pore state, a subscript is needed to indicate : **P**- the **P** site bound states (black states) that shift activation and enhances inactivation and, **W**- a separate **W**ithheld state (blue state), from which the IB region cannot directly access the pore block state. Rate constants k_c_(v)  = 40(−0.015) and k_o_(v)  = 1500(0.32) as described previously. Equilibrium constants and cooperativity factor α determined as described in the text. The model produces a reasonable fit for the steady state properties for AKv1, AKv1(I8Q), and AKv1(E2D) (with K_I_ changed to 0.1). Accurate activation and inactivation kinetics at strong depolarizations requires additional steps along the red pathway to rate limit the kinetics. If direct closing from O_P_ and O_W_ open states is included using the same k_c_(v) value then closing and recovery kinetics for AKv1, AKv1(I8Q), and AKv1(E2D) channels at strong negative potentials can be reproduced with this model if the K_I_ equilibrium is made rapid. B) Structural model of key regions involved in N-type inactivation based on the 3LUT structure of Kv1.2 [23]. A tilted perspective (see inset upper right) of the channel showing the inner aqueous volume of the channel in gray. Residue Tyr417 is shown in red and the S4-S5 linker in green. Selectivity filter is marked by the locations of the potassium ions in purple. C) Internal aqueous volume of the channel seen from a side perspective divided into the pore inner vestibule (white) and the side window vestibule (green). Volumes of these regions are given in the matching color. A single subunit P region backbone trace from the S4-S5 linker to the end of the determined S6 structure is shown along with its residue Tyr417 (red) and S4-S5 linker (brown) highlighted. The S4-S5 linker from the adjacent subunit is close to Tyr417 and therefore is also shown. D) Same picture only rotated 90^o^ to show the locations of Tyr417 and the S4-S5 linkers from 2 subunits relative to the pore inner vestibule and the side window vestibule. Both Tyr417 and the S4-S5 linkers are side window vestibule lining residues, not pore inner vestibule lining residues.

It is relatively easy to reproduce the basic equilibrium effects of IP region function on activation and inactivation by modifying the single step open and inactivation model shown in Fig. 1 to include a parallel state conforming to the IP region binding to its Pre-Block (P) site, see Fig. 9A. If the IP binding affinity (K_P_) is at least 2.6, then this binding on its own is sufficient to shift channel activation by the ∼ −4 mV produced by the IP region. However, IP region binding on its own is not sufficient to explain the difference between the apparent affinity of the IB region for the pore block site (K_I_) between AKv1 and AKv1(I8Q). Reproducing this effect requires that IP region binding has some positive cooperativity (α) with IB region binding into the pore (Fig. 9A). If the enhancement in binding affinity is at least 12 fold then the apparent K_I_ of 1.8 for AKv1(I8Q) would shift to approximately 21 as seen with AKv1. For AKv1(E2D) the inactivation properties can be closely approximated by retaining these values for K_P_ and α but changing the K_I_ to 0.1.

Finally, we noted that the fraction block at strong depolarizations seen with AKv1(I8Q) (0.48) is less than expected for K_I_ = 1.8 (expected 0.64) and that this channel closes with two exponentials. These observations suggest that a fraction of the channels exist in a state that is open but not subject to inactivation, a state we have named the **W**ithheld (**W**) state (Fig. 9A). If the equilibrium constant for N-terminal occupancy of the W state compared to the preferred state from which N-type inactivation normally proceeds is ∼1 then this would explain the mismatch in observed fractional block (1.8/(1+1+1.8)  = 0.47) as well as the rapid closing of these W state channels.

These studies have not characterized the nature of the hypothesized W state; however, there are a few likely possibilities. First, previous studies have found that there are hydrophobic interaction sites for the N-terminus along the side window connections to the pore that decrease the efficiency of inactivation by withholding the IB region of the N-terminus from the pore block site [11]. Second, the internal volume of the channel from the side window opening to the selectivity filter is over 11,000 Å^3^ (see Fig. 9B-D: 10,600 Å^3^ side window vestibule, 780 Å^3^ pore inner vestibule), compared to a peptide made from the first 9 residues of the N-terminus which has a solvent accessible excluded volume of 1,200 Å^3^. This means that entropic considerations alone should allow the N-terminus to occupy many configurations within this much larger channel internal volume. Those configurations that cannot directly lead to N-type inactivation would be seen as W states. Finally, specific binding interactions within the N-terminus itself, such as hair pin folds, rather than interactions with residues lining the side window vestibule could lead to W states that cannot directly inactivate. In reality, the W state may reflect some combination of all these effects, and their relative importance could be affected by mutations to the N-terminus.

For AKv1(I8Q) partial occupancy of the W state would also explain the double exponential closing kinetics for this channel. For AKv1 and AKv1(E2D), the occupancy of the W state would be much lower due to the effects of K_P_ and α drawing the N-terminus away from this site, producing single exponential closing kinetics. On the other hand, mutations that produce greater occupancy of the W state would produce a K_I_^1^ measure of inactivation affinity that greatly underestimates the true affinity of the pore block. Such an effect could help explain the poor correlation between Equation (1.2) and the inactivation curve midpoints for AKv1(E2x) mutants seen in Fig. 2 when using the K_I_^1^ estimate for K_I_. We have previously noted that some N-terminal mutants, such as AKv1(E2N), paradoxically have large sustained currents but recover slowly from inactivation [11]. This behavior could be explained by the model shown in Fig. 9A if the E2N mutation produces both a higher K_I_ and a higher K_W_.

As discussed in regards to different activation models, constructing a model that simply and efficiently reproduces the correct kinetics for all aspects of AKv1 and mutant inactivation gating is challenging. At this point it is unclear which specific activation states are capable of supporting IP region binding to the P site, how complex the transitions steps are between the fully free state for the N-terminus and binding to the P site, which of these transitions can be crossed at a particular level of channel activation, and whether the similarity in voltage dependence between closing and recovery reflects a rapid inactivation pore block/unblock process or a voltage dependent tugging of the ball from the pore [17], or some other process. A key future experiment is to more closely examine the inactivation and recovery cycle by single channel methods to determine if there are significant changes in apparent single channel conductance that are dependent on ball peptide apparent affinity, reflecting rapid pore block/unblock, or whether the Camacho tugging model provides a better description of the channel recovery process.

### Constraining the IP motif binding site and its Importance for Ion Channel Function

Obviously an important question for future studies is to identify the precise binding site within the channel that is responsible for the forming the P state interaction with the IP region. Our data suggest that the IP region binds within the side window vestibule (Fig. 9B-D) at a site near the pore, but not directly within the pore, to enhance the ability of the N-terminus to block the channel: 1) The IP region does not block conductance therefore it is unlikely to bind with the pore (Fig. 6A). 2) Channel closing is not slowed by the IP region binding (Fig. 6D); therefore, it most likely binds outside the region occluded by the activation gate. 3) The volume of the pore inner vestibule (780 Å^3^) is smaller than the residue 1-7 peptide excluded volume preceding residue Ile8 (870 Å^3^) and similar to the contact surface volume for this segment of (673 Å^3^); therefore, Ile8 is most likely outside the pore inner vestibule in the N-type inactivated state (Fig. 9B-D). 4) Previous studies have suggested that residue 2 in the AKv1 N-terminus binds deeply within the pore vestibule in the inactivated state as has been proposed for other IB N-terminal sequences [11], [13], [22]. The distance from the selectivity filter to the side window openings is ∼40 Å, so even with a fully extended structure Ile8 is most likely within the side window vestibule when the channel is N-type inactivated (3.5 Å×8 = 28 Å). 5) Mutant cycle analysis on the rat KCNAB1 inactivation domain region showed a strong interaction between residue Ile5 and a tyrosine residue that aligns with Tyr417 in the Kv1.2 structure [10], [13], [23]. Although originally proposed to be part of the pore inner vestibule, in the determined structure Tyr417 is clearly a side window vestibule lining residue (Fig. 9B-D).

The alignment of N-termini in Fig. 7A suggests that rat KCNAB1 residue Ile5 might function as an H_5_ residue in this subunit’s IP motif, which might point towards Kv1.2 residue Tyr417 as a possible location for the IP region binding site. However, even if this alignment is misleading and Met5 in AKv1 actually binds to the residue homologous to Tyr417, then the IP motif binding site is likely within 10−15 Å of this residue in the direction of the side window openings. Within this target region there are two conserved regions of the channel that could help form a binding pocket: the distal end of the S6 transmembrane domain, and the S4-S5 linker. Interestingly, previous studies have suggested that the S4-S5 linker plays an important role in N-type inactivation [24], [25], even though it is clearly located well outside the pore inner vestibule. Our future studies will specifically test these sites to determine if they form a hydrophobic binding pocket for the H_5_ residue of the IP motif.

### General Importance of the Pre-Block Interaction Site

The effects on the IP domain on channel activation have revealed a novel regulatory phenomenon that could have much broader implications for understanding the regulation of ion channel function. Although the shift in activation produced by the IP domain is rather modest, amounting to a few mV, even such a small shift could have important implications for channel function *in vivo* since the activation curve for the channel is very steep. It is also possible that other N-termini on channels, or auxiliary subunits, might have evolved to produced much larger effects on activation by binding to this site as their primary function [26]. It is also important to note that the Pre-Block binding site for the IP motif is a potentially important new target for drug discovery, since it can modulate both inactivation and activation. In addition, this binding pocket may play an important role in the function of known drugs and N-terminal domains that modulate potassium activation or inactivation without directly causing pore block [27], [28], [29], [30]. Finally, an important future question is the extent to which regulation of N-type inactivation by phosphorylation, re-dox, RNA editing, or other modifications is due to disruption of the IP region’s interaction with the P site and whether dynamic regulation of N-terminal effects on activation through this modulatory site is a common phenomenon [22], [31], [32], [33].

## Materials and Methods

### Ethics Statement

The procedures on animals conducted in this work were performed in strict accordance with Animal Welfare Act, the Public Health Services Animal Welfare Policy, and The National Institute of Health Guide for Care and Use of Laboratory Animals. The experimental protocol was approved by the Institutional Animal Care and Use Committees (IACUC) of Baylor College of Medicine (Protocol Number: AN-752). Following the approved protocol, every effort was made to minimize suffering.

### Molecular Methods

AKv1 and AKv1(E2x) mutant channels were described previously [11], [12]. Point mutations and deletions were constructed by PCR based methods and confirmed by DNA sequencing of both strands. Messenger RNA was synthesized using the Message Machine kit (Ambion). Homology analysis and alignments were performed with Jalview [34], [35].

### Electrophysiology Methods and Data Analysis

**Recordings.** Currents were recorded from *Xenopus* oocytes 1−3 days after injection of mRNA as described previously [11], [12]. Steady state inactivation was measured by first determining the time to reach a steady state, then using a holding time at least this long prior to performing a test pulse. Typically this hold time was at least 20x as long as the time constant to recover from inactivation at −100 mV. Recordings were performed in elevated extracellular K^+^ to reduce the effects of C-type inactivation [12]. The normal Hi K bath solution was: (in mM: KCl 98, MgCl2 1, CaCl_2_ 1.8, and HEPES 5 at pH 7.4). Recording electrodes were pulled on a Sutter Instruments P-97 puller to a resistance of 0.5–1 MΩ and were backfilled with 3 M KCl.

**Data Analysis.** Capacitance and leak currents were removed by off line P/5 leak subtraction. Data analyses were performed with WinWCP (John Dempster, University of Strathclyde), pClamp10 (Molecular Devices), Origin 6.1 (Origin Labs), GraphPad (GraphPad Software) and Excel (Microsoft) as described previously. Data are reported as reported as the mean ± SEM (n = number of independent measurements). Significance testing was performed using unpaired two tailed t-tests comparing to wild type, unless otherwise indicated. Measured P values are reported, with the significance level set at 0.05. Results that are not significantly different are indicated by NSD.

**Modeling Single Step Boltzmann Inactivation Curve.** Consider a voltage gated channel with a single step N-type inactivation mechanism. Assume a voltage dependent equilibrium constant exists for the Open state at any given voltage (K_O_(V)) and a non-voltage dependent equilibrium constant exists for the Inactivated state(K_I_). Then we can write the following reaction diagram:



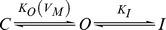



We can see that the probability of being inactivated at any membrane potential at the end of a long pulse is:
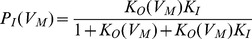



The largest fraction of channels that can be inactivated given a very strong depolarization such that, P_C_→ 0, is:
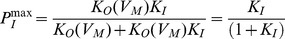



Using this as a normalizer, we can define the Boltzmann curve for the fractional probability of being inactivated at any membrane potential relative to the maximum possible inactivation as:
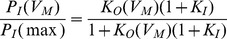



**Modeling Relationship between Steady State Inactivation and Recovery Rate.** Assume the following model for recovery from Inactivation at strong negative potentials with the K_I_ equilibrium fast relative to k_c_:
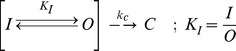



Using probabilities for the three different states and the fact the I and O states remain in virtual equilibrium:
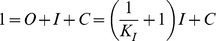



Differentiating with respect to time:



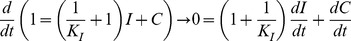
(1.5)


From the Reaction diagram we see:



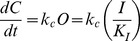
(1.6)


Substituting Equation (1.6) into Equation (1.5), we get:



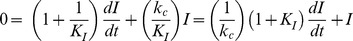
(1.7)


Thus the time constant to recover from inactivation is:



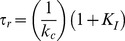
(1.8)


The time constant to close for this channel without N-type inactivation is:



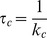
(1.9)


Therefore:



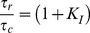
(1.10)


**Energetics and Computational modeling.** Computational modeling of AKv1 gating was performed with QuB (SUNY Buffalo) using the MacRates module [36]. For single step activation modeling, the general approach was to fix the closing rate constant to match the observed data and then adjust the opening rate constant to produce the correct overall voltage dependence. Rate constants are described by two constants and written k_0_(v_k_) corresponding to the formula: rate  = k_0_*exp(v_k_*E_M_) with k_0_ in units of sec^−1^ and v_k_ mV^−1^. For the single step Activation model, the rate constants used were: k_c_(v)  = 40(−0.015) and k_o_(v)  = 1500(0.32). For the Aldrich type activation model, the class D model from Fig. 7 of the Shaker Potassium Channel Gating III paper [20] was used with parameters: α = 250(0.02); β = 1000(−0.091); γ = 2800 (0.041); δ = 85 (−0.015) ; θ = 8.5. Equilibrium constants defined as: K_I_ = I/O; K_P_ = O_P_/O; K_W_ = O_W_/O. Energetics for hydrophobic burial of an Isoleucine side chain from Karplus (1997) [21]. Energetics calculations for the effects of Ile8 binding on activation and inactivation were performed using the following equations: 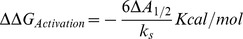


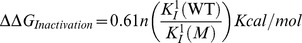



Structural modeling was performed using the Kv1.2 3LUT channel model [23] based on normal mode refinement of the MacKinnon lab original Kv1.2 structure 2A79 [10]. Channel inner aqueous volume was determined using the ^3^V server with the Channel Finder function (3vee.molmovdb.org) [37] and visualized with UCSF Chimera [38] and VMD [39]. N-terminal peptide excluded volume was calculated using the ^3^V server with the Volume Assessor function using a 1.5 Å probe radius, and contact surface volume with a 0.0 Å probe radius. Zhou et al. (2001) used mutant cycle analysis to identify an interaction between rat KCNAB1 residue Ile5 and Kv1.4 channel residue Tyr569 [13]. Residue Tyr569 from Kv1.4 (CAA34133) was confirmed to align with residue Tyr417 from the 3LUT structure using Jalview [34], [35].

## References

[1] Hille (2001) Ion Channels of Excitable Membranes. SunderlandMA: Sinauer Associates Inc. 814 p.

[2] Rasmusson RL, Morales MJ, Wang S, Liu S, Campbell DL, et al (1998) Inactivation of voltage-gated cardiac K+ channels. Circ Res 82: 739–750. 9562433 10.1161/01.res.82.7.739

[3] Barros F, Dominguez P, de la Pena P (2012) Cytoplasmic domains and voltage-dependent potassium channel gating. Front Pharmacol 3: 49. 22470342 10.3389/fphar.2012.00049 PMC3311039

[4] Fineberg JD, Ritter DM, Covarrubias M (2012) Modeling-independent elucidation of inactivation pathways in recombinant and native A-type Kv channels. J Gen Physiol 140: 513–527. 23109714 10.1085/jgp.201210869 PMC3483116

[5] Hoshi T, Zagotta WN, Aldrich RW (1990) Biophysical and molecular mechanisms of Shaker potassium channel inactivation [see comments]. Science 250: 533–538. 2122519 10.1126/science.2122519

[6] Aldrich RW (2001) Fifty years of inactivation. Nature 411: 643–644. 11395746 10.1038/35079705

[7] Zagotta WN, Aldrich RW (1990) Voltage-dependent gating of Shaker A-type potassium channels in Drosophila muscle. J Gen Physiol 95: 29–60. 2299331 10.1085/jgp.95.1.29 PMC2216290

[8] Murrell-Lagnado RD, Aldrich RW (1993) Interactions of amino terminal domains of Shaker K channels with a pore blocking site studied with synthetic peptides. J Gen Physiol 102: 949–975. 8133245 10.1085/jgp.102.6.949 PMC2229190

[9] Long SB, Tao X, Campbell EB, MacKinnon R (2007) Atomic structure of a voltage-dependent K+ channel in a lipid membrane-like environment. Nature 450: 376–382. 18004376 10.1038/nature06265

[10] Long SB, Campbell EB, Mackinnon R (2005) Crystal structure of a mammalian voltage-dependent Shaker family K+ channel. Science 309: 897–903. 16002581 10.1126/science.1116269

[11] Prince A, Pfaffinger PJ (2013) Conserved N-terminal negative charges support optimally efficient N-type inactivation of Kv1 channels. PLoS One 8: e62695. 23638135 10.1371/journal.pone.0062695 PMC3634772

[12] Prince-Carter A, Pfaffinger PJ (2009) Multiple intermediate states precede pore block during N-type inactivation of a voltage-gated potassium channel. J Gen Physiol 134: 15–34. 19528261 10.1085/jgp.200910219 PMC2712980

[13] Zhou M, Morais-Cabral JH, Mann S, MacKinnon R (2001) Potassium channel receptor site for the inactivation gate and quaternary amine inhibitors. Nature 411: 657–661. 11395760 10.1038/35079500

[14] Lee TE, Philipson LH, Nelson DJ (1996) N-type inactivation in the mammalian Shaker K+ channel Kv1.4. J Membr Biol 151: 225–235. 8661510 10.1007/s002329900073

[15] Gonzalez-Perez V, Zeng XH, Henzler-Wildman K, Lingle CJ (2012) Stereospecific binding of a disordered peptide segment mediates BK channel inactivation. Nature 485: 133–136. 22522931 10.1038/nature10994 PMC3348258

[16] Demo SD, Yellen G (1991) The inactivation gate of the Shaker K+ channel behaves like an open-channel blocker. Neuron 7: 743–753. 1742023 10.1016/0896-6273(91)90277-7

[17] Camacho CJ (2008) Quantitative modeling of currents from a voltage gated ion channel undergoing fast inactivation. PLoS One 3: e3342. 18833322 10.1371/journal.pone.0003342 PMC2551740

[18] Pfaffinger PJ, Furukawa Y, Zhao B, Dugan D, Kandel ER (1991) Cloning and expression of an Aplysia K+ channel and comparison with native Aplysia K+ currents. J Neurosci 11: 918–927. 2010814 10.1523/JNEUROSCI.11-04-00918.1991 PMC6575383

[19] Murrell-Lagnado RD, Aldrich RW (1993) Energetics of Shaker K channels block by inactivation peptides. J Gen Physiol 102: 977–1003. 8133246 10.1085/jgp.102.6.977 PMC2229186

[20] Zagotta WN, Hoshi T, Aldrich RW (1994) Shaker potassium channel gating. III: Evaluation of kinetic models for activation. J Gen Physiol 103: 321–362. 8189208 10.1085/jgp.103.2.321 PMC2216839

[21] Karplus PA (1997) Hydrophobicity regained. Protein science : a publication of the Protein Society 6: 1302–1307. 9194190 10.1002/pro.5560060618 PMC2143722

[22] Gonzalez C, Lopez-Rodriguez A, Srikumar D, Rosenthal JJ, Holmgren M (2011) Editing of human K(V)1.1 channel mRNAs disrupts binding of the N-terminus tip at the intracellular cavity. Nat Commun 2: 436. 21847110 10.1038/ncomms1446 PMC3265383

[23] Chen X, Wang Q, Ni F, Ma J (2010) Structure of the full-length Shaker potassium channel Kv1.2 by normal-mode-based X-ray crystallographic refinement. Proceedings of the National Academy of Sciences of the United States of America 107: 11352–11357. 20534430 10.1073/pnas.1000142107 PMC2895106

[24] Holmgren M, Jurman ME, Yellen G (1996) N-type inactivation and the S4-S5 region of the Shaker K+ channel. Journal of General Physiology 108: 195–206. 8882863 10.1085/jgp.108.3.195 PMC2229322

[25] Isacoff EY, Jan YN, Jan LY (1991) Putative receptor for the cytoplasmic inactivation gate in the Shaker K+ channel. Nature 353: 86–90. 1881453 10.1038/353086a0

[26] Jerng HH, Pfaffinger PJ (2012) Incorporation of DPP6a and DPP6K variants in ternary Kv4 channel complex reconstitutes properties of A-type K current in rat cerebellar granule cells. PLoS One 7: e38205. 22675523 10.1371/journal.pone.0038205 PMC3366920

[27] Roeper J, Sewing S, Zhang Y, Sommer T, Wanner SG, et al (1998) NIP domain prevents N-type inactivation in voltage-gated potassium channels. Nature 391: 390–393. 9450755 10.1038/34916

[28] Holmqvist MH, Cao J, Hernandez-Pineda R, Jacobson MD, Carroll KI, et al (2002) Elimination of fast inactivation in Kv4 A-type potassium channels by an auxiliary subunit domain. Proc Natl Acad Sci U S A 99: 1035–1040. 11805342 10.1073/pnas.022509299 PMC117799

[29] Lu Q, Peevey J, Jow F, Monaghan MM, Mendoza G, et al (2008) Disruption of Kv1.1 N-type inactivation by novel small molecule inhibitors (disinactivators). Bioorg Med Chem 16: 3067–3075. 18226531 10.1016/j.bmc.2007.12.031

[30] Zhang ZH, Rhodes KJ, Childers WE, Argentieri TM, Wang Q (2004) Disinactivation of N-type inactivation of voltage-gated K channels by an erbstatin analogue. The Journal of biological chemistry 279: 29226–29230. 15136567 10.1074/jbc.M403290200

[31] Beck EJ, Sorensen RG, Slater SJ, Covarrubias M (1998) Interactions between multiple phosphorylation sites in the inactivation particle of a K+ channel. Insights into the molecular mechanism of protein kinase C action. J Gen Physiol 112: 71–84. 9649584 10.1085/jgp.112.1.71 PMC2229409

[32] Ruppersberg JP, Stocker M, Pongs O, Heinemann SH, Frank R, et al (1991) Regulation of fast inactivation of cloned mammalian IK(A) channels by cysteine oxidation. Nature 352: 711–714. 1908562 10.1038/352711a0

[33] Drain P, Dubin AE, Aldrich RW (1994) Regulation of Shaker K+ channel inactivation gating by the cAMP- dependent protein kinase. Neuron 12: 1097–1109. 8185946 10.1016/0896-6273(94)90317-4

[34] Waterhouse AM, Procter JB, Martin DM, Clamp M, Barton GJ (2009) Jalview Version 2—a multiple sequence alignment editor and analysis workbench. Bioinformatics 25: 1189–1191. 19151095 10.1093/bioinformatics/btp033 PMC2672624

[35] Clamp M, Cuff J, Searle SM, Barton GJ (2004) The Jalview Java alignment editor. Bioinformatics 20: 426–427. 14960472 10.1093/bioinformatics/btg430

[36] Milescu LS, Akk G, Sachs F (2005) Maximum likelihood estimation of ion channel kinetics from macroscopic currents. Biophys J 88: 2494–2515. 15681642 10.1529/biophysj.104.053256 PMC1305347

[37] Voss NR, Gerstein M (2010) 3V: cavity, channel and cleft volume calculator and extractor. Nucleic acids research 38: W555–562. 20478824 10.1093/nar/gkq395 PMC2896178

[38] Pettersen EF, Goddard TD, Huang CC, Couch GS, Greenblatt DM, et al (2004) UCSF Chimera—a visualization system for exploratory research and analysis. Journal of computational chemistry 25: 1605–1612. 15264254 10.1002/jcc.20084

[39] Humphrey W, Dalke A, Schulten K (1996) VMD: visual molecular dynamics. J Mol Graph 14: 33−38, 27−38. 10.1016/0263-7855(96)00018-5 8744570

